# Influence of Particle Size and Xylanase in Corn-Soybean Pelleted Diets on Performance, Nutrient Utilization, Microbiota and Short-Chain Fatty Acid Production in Young Broilers

**DOI:** 10.3390/ani10101904

**Published:** 2020-10-17

**Authors:** Diego Melo-Durán, José Francisco Pérez, Gemma González-Ortiz, Roser Sala, Sandra Villagómez-Estrada, Michael R. Bedford, Hadden Graham, David Solà-Oriol

**Affiliations:** 1 Animal Nutrition and Welfare Service (SNIBA), Department of Animal and Food Science, Universitat Autonòma de Barcelona, 08193 Bellaterra, Spain; diego.melo.d@outlook.com (D.M.-D.); josefrancisco.perez@uab.cat (J.F.P.); roser.sala@uab.cat (R.S.); sandra.villagomez@outlook.es (S.V.-E.); 2 AB Vista, Marlborough, Wiltshire SN8 4AN, UK; gemma.gonzalez@abvista.com (G.G.-O.); mike.bedford@abvista.com (M.R.B.); hg@foliumscience.com (H.G.)

**Keywords:** microbiota, nutrient digestibility, particle size, short-chain fatty acids, xylanase

## Abstract

**Simple Summary:**

The use of enzymes, such as xylanase, in poultry production has the objective of improving the productive value of diets by releasing nutrient content and boosting animal performance, with the consequent reduction in feed costs and environmental impact. Additionally, xylanase could have benefits in the intestinal microbiota through the prebiotic effect of oligosaccharides produced from the arabinoxylans present in the diet. In broilers, little is known of how feed processing of the diet, such as pelletizing, particle size, and grinding method, could affect enzyme efficacy in corn-based diets. The present study aimed to understand the effects of corn particle size and xylanase supplementation in pelleted diets on the performance, nutrient utilization, short-chain fatty acid concentration, gut microbiota, and intestinal development of young broilers. Our results have shown evidence of the importance of coarse particles on the gut development and digestibility of nutrients, and along with the xylanase, has shown benefits on microbiota modulation and in reducing the variation in body weight.

**Abstract:**

The objective of this study was to investigate the effects of particle size and xylanase supplementation in corn-based pellet diets on the performance and digestive traits in young broilers. A total of 512 male Ross 308 broilers were used in a 21-day study. The treatments were designed in a 4 × 2 factorial arrangement with four levels of geometric mean diameter (D_gw_) of corn (540, 660, 1390, and 1700 µm), and two levels of xylanase (0 or 16,000 BXU/kg diet). Feeding coarse corn diets (1390 and 1700 µm D_gw_) and xylanase supplementation showed an inferior coefficient of variation of body weight. Higher gizzard weight, microbiome alpha-diversity, and clustered separately beta-diversity (*p* < 0.05) were observed in birds fed coarse diets. The addition of xylanase promoted changes in relative bacteria abundance, increasing Lachnospiraceae, Defluviitaleaceae, Bacteroidaceae, Bacillaceae, Eggerthellaceae, and Streptococcaceae families in the 1700 µm group, and Christensenellaceae and Lachnospiraceae families in the 540 µm D_gw_ group. In conclusion, xylanase supplementation and particle size of corn interact in the intestinal environment, showing changes in microbial composition. Coarse diets and xylanase supplementation showed improved body weight homogeneity, which might be related to a better gut development and microbiota modulation.

## 1. Introduction

The main objective of grinding poultry feed ingredients is to crush the grain cell wall and increase the surface for the digestive enzymes; however, it is important to obtain an optimal particle size distribution (PSD). The PSD needs to be adapted to the physiological capabilities of the birds in order to improve nutrient digestibility and animal performance, and to facilitate further feed processing, such as mixing, pelleting, or extrusion/expansion [1,2]. In poultry diets, coarse particles play a physical role in gut development and function, increasing the size of digestive organs such as the gizzard [3], and the retention time in the proximal portion of the digestive tract. In this way, positive effects, such as improved nutrient digestibility and reduced litter moisture content, have been described with coarse grain particle diets, either with corn or barley [4,5,6,7]. In Europe, grinding ingredients for poultry diets are mostly undertaken with hammer mills, usually fitted with a screen between 3 and 4.5 mm in size [8], and commonly presented in a pellet form, since the benefits are greater than in mash presentation. However, pelletizing applies an additional grinding of particles (secondary grinding), which is mainly caused by rollers of the pellet press [9]. This increases the extra micro-particles and reduces the percentages of coarse particles [10]. In addition to their effects on gut development of chickens, particle size and form might also influence the activity of digestive enzymes based on their different surface area. Xylanase is commonly added when viscous cereals, such as wheat, are used in poultry diets in order to reduce digesta viscosity of birds by degradation of soluble arabinoxylans [11]. Moreover, xylanase also shows benefits when used in corn diets [12,13], due to the disruption of the cell wall, which releases encapsulated nutrients (“cage effect”), and/or through the prebiotic effect of xylo-oligosaccharides (XOS) or arabinoxylo-oligosaccharides (AXOS) produced from corn arabinoxylans [14,15,16]. In broiler diets, the efficacy of supplemental xylanase was influenced by the wheat particle size on pellet diets [17], being enhanced in the presence of whole wheat [18]. However, with regard to the degree of grain grinding in corn base diets, there are no previous studies examining the interaction between xylanase supplementation and corn particle size in poultry diets. Thus, we hypothesize that the coarse corn particles could improve the response of xylanase supplementation in corn-based diets. Therefore, the aim of the present study was to investigate the effects of particle size and xylanase supplementation on the performance, nutrient utilization, short-chain fatty acid concentration, gut microbiota, and intestinal morphology of young broilers fed with corn-based pelleted diets.

## 2. Materials and Methods

### 2.1. Ethics Statement

The experimental procedures were approved by the Animal Experiment Committee (CEEAH) of the Universitat Autònoma de Barcelona (number code: CEEAH 10167), and were in compliance with the European Union guidelines for the care and use of animals in research [19].

### 2.2. Birds and Housing

A total of 512 Ross 308 day-old male broilers were purchased from a local hatchery. Upon arrival, birds were individually weighed and assigned to 64 wire-floored battery cages with eight birds per cage. Each cage (62 cm × 48 cm × 37 cm) was equipped with a line feeder and a nipple waterer. Test diets and water were provided ad libitum throughout the trial. The room was environmentally controlled and pre-heated to 34 °C two days prior to the start of the study. During the first two days, the temperature was set at 32 °C, and was gradually reduced to 20 °C at the end of the trial (d 21). The birds were given 24 h of light for the first 2 days, which was reduced to 23 h of light and 1 h of dark from d 3 to d 10, and 18 h of light and 6 h of dark from d 11 until the end of the experimental period.

### 2.3. Experimental Diets

The experimental design was a 4 × 2 factorial arrangement, consisting of four levels of geometric mean particle size diameter (D_gw_) for corn (540, 660, 1390 and 1700 µm), and two levels of xylanase supplementation, 0 or 100 g/ton of xylanase (Econase XT 25P, AB Vista, Marlborough, UK; 160,000 BXU/g). Corn geometric mean diameter (D_gw_) was measured in the feed-mill, prior to the pelleting process, using the procedure of three-sieve analysis [20]. Experimental diets (Table 1) were offered in pellet form, and met the nutrient recommendations for broilers from d 0 to d 21 [21]. Corn was obtained from a commercial batch at a local feed-mill. The four particle sizes of corn (540, 660, 1390, and 1700 µm) were obtained using a horizontal hammer mill with different configurations, corresponding to 1500, 1200, 600, and 450 rpm mill speed, and 5, 5, 8, and 8 mm mill screen size, respectively. The soybean meal for all diets was milled at the same configuration of 540 µm D_gw_ (speed 1500 rpm; screen size 5 mm). The diets were cold pelleted (70 °C) using a pellet press (Pellet Mill 3020-4) with a capacity of 4500–5500 kg/h, and using a 2.2 mm die. The particle size spectrum was characterized by dry sieving using a method described by Baker and Herrman [22] for corn, soybean meal and mash diets, and wet sieving using a method described by Miladinovic [23] for pelleted diets. The D_gw_ and geometric standard deviation (S_gw_) were then determined. Diets contained 0.5% titanium dioxide as an indigestible marker for the estimation of nutrient digestibility.

### 2.4. Experimental Procedures

Birds were weighed individually on d 0 and 21. Body weight (BW) uniformity was expressed as the coefficient of variation (CV) of BW. Feed consumption was determined by cage, and mortality was monitored twice daily. The weights of dead birds were used to adjust the feed conversion ratio (FCR). From d 19, excreta samples from all cages were collected over a 48-h period using aluminum trays. Feather down and feather scales were removed. At the end of the collection period, the excreta were collected in plastic containers, immediately frozen at −20 °C, and then freeze dried. On d 21, three birds from each of the 64 cages were randomly selected and euthanized by cervical dislocation. The complete gastrointestinal tract (GIT) was removed immediately from the abdominal cavity and dissected. The empty weights of the total GIT and each of its sections were recorded. Digesta contents from the ileum and cecum were collected separately on a cage basis by gently squeezing the contents out of the relevant sections from the three animals per cage, and pooled. The ileal digesta and excreta were ground to pass through a 0.5 mm screen in a grinder, before the analysis for apparent nutrient digestibility measurements. Cecal contents were analyzed for short chain fatty acid (SCFA) concentration. An additional aliquot of cecal contents from the 540 and 1700 µm treatments (with and without xylanase inclusion) were frozen at −80 °C for microbiota 16S rRNA gene sequence analysis.

### 2.5. Samples Analyses

The contents of dry matter (DM), ash, crude protein (CP) and gross energy (GE) were analyzed in feed, ileal digesta and excreta. Additionally, starch analyses were performed according to the 996.11 of Association of Official Analytical Chemists (AOAC) International (2000) method for both feed and ileal contents. Diet proximate analyses were performed according to the AOAC (2005) [24] Official Methods: Method 968.06 (CP), Method 934.01 (DM), Method 942.05 (ash), and GE was determined by using an isoperibolic calorimeter (Parr, Parr Instrument Company, Moline, Illinois, USA). Activity of xylanase was determined using the method of analysis recommended by the supplier. Xylanase activity was determined at pH 5.3 and 50 °C, using birchwood xylan as a substrate.

Apparent ileal and total tract digestibility of CP, GE, DM and organic matter (OM) were calculated by the index method using the following equation:

(1) $$Ileal\;and\;total\;tract\;apparent\;digestibility\;=((Ti_{D}/Ti_{M})/(N_{M}/N_{D}))$$

where *Ti_D_* is the concentration of the Titanium (*Ti*) in the diet, *Ti_M_* is the concentration of *Ti* in ileal digesta or excreta, *N_M_* is the nutrient content in ileal digesta or excreta and *N_D_* is the nutrient content in the diet.

The estimate of total intake of digestible nutrients was obtained using the coefficients of digestibility, diet nutrient composition, and total feed intake.

### 2.6. Pellet Quality

Pellet quality was determined as pellet durability index (PDI), which measures the portion of fines generated during standardized mechanical handling, using Holmen pellet tester (NHP 100, Norfolk, UK). Duration of treatment was 30 s, and fines were removed before and after the treatment using the sieve with 2 mm openings diameter.

### 2.7. Dry Matter Solubility and Water Retention Capacity

Solubility of the DM (SolDM) and water retention capacity (WRC) for all diets were evaluated (*n* = 7). Samples were treated following an in vitro procedure that simulates gastric and small intestine pH [25], separately. In short, 0.5 g per duplicate of each sample was weighed into 10 mL screw cap tubes and incubated with 5 mL of 0.1 M sodium phosphate buffer (Na_2_HPO_4_) and 2 mL of 0.2 M hydrochloric acid (HCl) to simulate gastric pH (pH = 2.5), and 7 mL of 0.1 M sodium phosphate buffer to simulate small intestine pH (pH = 5). Tubes were kept at 41 °C for 120 min in a horizontal shaking water bath. The final volume of liquid was 7 mL both in the gastric and in the small intestine incubation. The amount of sample submitted for analysis was recorded (*W*0), as well as the weight of the screw cap tube plus sample (*W*1). After incubation, WRC was measured by centrifugation for 20 min at 2000× *g*. The supernatant was carefully removed, and the tubes were kept upside down for 10 min to ensure that the non-retained water was drained. Tubes with the sample were then weighed (*W*2), and dried in the oven at 100 °C for 16 h to ensure the complete drying of the insoluble residue, and then weighed again (*W*3). The solubility of the dry matter was calculated as follows:

(2) $$SolDM=\frac{W1-W3}{W0}$$

Water retention capacity determined after centrifugation is expressed as the gram of water retained by the total amount of sample incubated:

(3) $$WRC=\frac{W2-W3}{W0}$$

### 2.8. Short-Chain Fatty Acids

The SCFA in the ceca was analyzed as free acids by gas chromatography using pivalic acid as an internal standard [26]. Briefly, one mL of H_2_O was mixed with 1 g of cecal content, and then one mL of 20 mM pivalic acid solution was added as an internal standard. After mixing, one mL of perchloric acid was added and the SCFA were extracted by shaking the mixture for 5 min. After centrifugation, perchloric acid in the supernatant was precipitated by adding 50 μL of 4 M KOH in 500 μL of supernatant. After 5 min, saturated oxalic acid was added and the mixture incubated at 4 °C for 60 min and then centrifuged. Samples were analyzed by gas chromatography using a glass column packed with 80/120 Carbopack B-DA/4% Carbowax 20M stationary phase (Supelco, Bellefonte, PA, USA), using helium as the carrier gas and a flame ionization detector. The acids measured were acetic, propionic, butyric, valeric, iso-butyric, 2-methyl-butyric, iso-valeric, and lactic acid.

### 2.9. Microbial Diversity Analysis

The DNA extracted from cecal samples was subjected to 16S ribosomal RNA gene sequence-based analysis to examine the profile of the bacterial communities. The V3-V4 region of the bacteria 16S ribosomal RNA gene were amplified by PCR (95 °C for 3 min, followed by 25 cycles at 95 °C for 30 s, 55 °C for 30 s, and 72 °C for 30 s and 72 °C for 5 min) using primers F5′-barcode-TCGTCGGCAGCGTCAGATGTGTATAAGAGACAGCCTACGGGNGGCW GCA G-3′ and R5′-GTCTCGTGGGCTCGGAGATGTGTATAAGAGACAGGACTACHVGGGTATCTAATCC-3′. A negative control of the DNA extraction was included, as well as a positive Mock Community control, to ensure quality control. After 25 cycles of amplifications, 550 pb amplicons were obtained. The Illumina Miseq sequencing 300 × 2 approach was used. Raw sequencing reads were quality clipped, assembled, and compared with available genomic sequences in the databases using proprietary Software, and were validated and subsequently completed with the Kraken Metagenomics [27] and QIIME [28] software. Taxonomic assignment of phylotypes was performed using a Bayesian Classifier trained with Silva database version 132 (99% OTUs full-length sequences [29].

### 2.10. Statistical Analysis

Individual weights were used to calculate the CV of BW within each cage. Normal distribution and homoscedasticity of variances was checked prior to the analysis by using the Shapiro-Wilk test and Levene’s test for UNIVARIATE and General Linear Model (GLM) procedures, respectively. Cage was the experimental unit for all other variables. Performance, SCFA, solDM, WRC, relative organ weights and coefficients of digestibility were analyzed as a factorial arrangement, using a 2-way ANOVA to identify any interaction between particle size and xylanase inclusion, with PROC GLM (SAS 9.4, SAS Institute Inc., Cary, NC, USA). In addition, orthogonal polynomial contrasts were used to test the linear and quadratic effects of increasing levels of particle size of the diets in PDI. Biostatistical analysis for microbiota was performed in open source software R-Studio v.3.6.1. (Boston, MA, USA). Diversity was analyzed at operational taxonomic units (OTU) level using a vegan package [30]. Richness and alpha diversity were calculated with raw counts based on Simpson, Shannon and Inverse-Simpson estimators. Beta diversity was evaluated by multivariate ANOVA based on dissimilarities, using the adonis function. Finally, differential abundance analysis was performed with taxa relative abundances under a zero-inflated log normal mixture model, *p*-values were corrected by false-discovery rate (FDR) using the metagenome Seq package [31]. Due to factorial arrangement, the main effects are discussed for responses in which the interaction was not significant. Significantly different means were separated using Tukey’s Honestly Significant Difference (HSD) test. Significance was declared at a probability *p* ≤ 0.05 and tendencies were considered when *p*-value was between >0.05 and <0.1.

## 3. Results

### 3.1. Feed Analyses and Diet Particle Size Analysis

**Figure 1:**
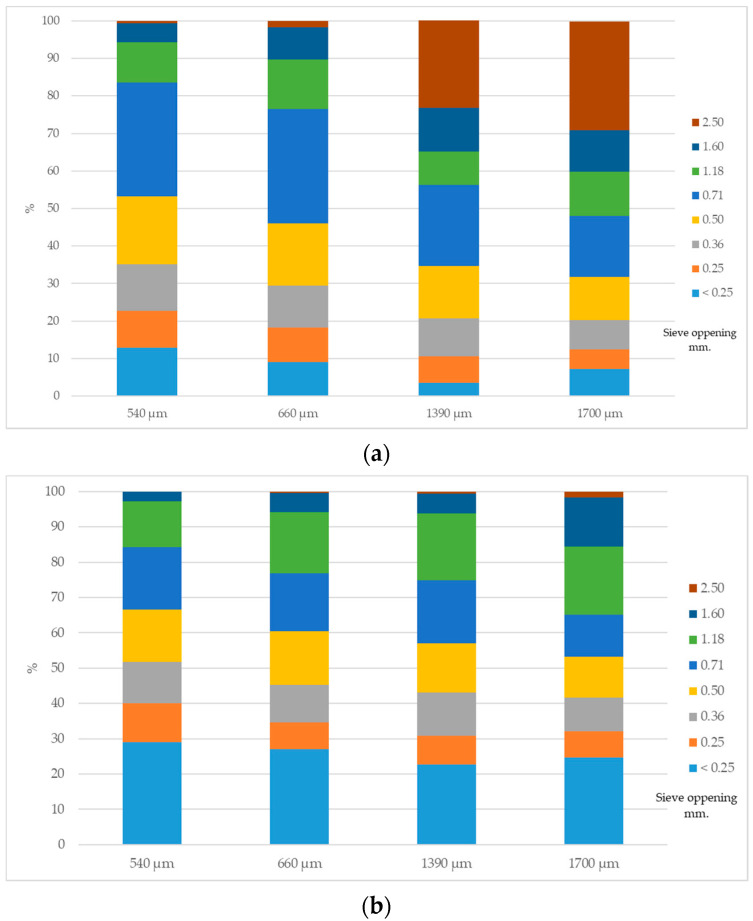
Particle size distribution of the three particle sizes of the mash (**a**) and pelleted diets (**b**).

The measurements of the nutrient composition (Table 1) and enzyme activities (Table 2) of all diets were all close to expected. Graphic comparisons of the particle size distributions of mash and pelleted diets (Figure 1) obtained by wet sieving, showed that pelleting reduced the relative proportion of coarse particles (>1000 µm) and increased the proportion of fine particles (<500 µm) in all diets. Geometric mean diameter (D_gw_) of pelleted diets manufactured using a ground corn with D_gw_ 540, 660, 1390, and 1700 µm were determined to be 488, 549, 583, and 637 µm, respectively, with corresponding standard deviation (S_gw_) values of 2.03, 2.13, 2.11, and 2.32 µm (Table 3).

### 3.2. Pellet Quality, Solubilization of Dry Matter and Water Retention Capacity

**Figure 2:**
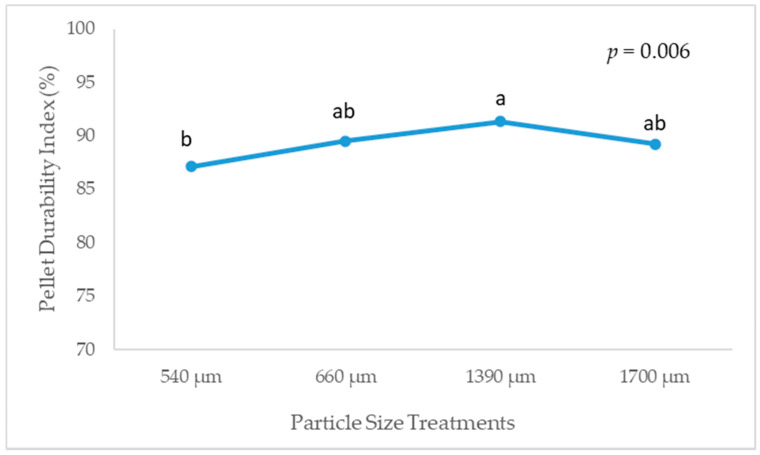
Effect of corn particle size on the pellet durability index (*n* = 4). ^a,b^ Treatments with different letters are statistically different (*p* < 0.05).

Pellet quality, determined by PDI, differed (linear, *p* = 0.014; quadratic, *p* = 0.006; Figure 2) due to mash particle size, with 540 µm D_gw_ having an inferior PDI (87.1%) than 1390 µm (91.3%); 660 and 1700 µm D_gw_ corn diets had intermediate values (89.5% and 89.2%, respectively).

The SolDM and WRC results after the in vitro incubation at pH 2 or 5 are shown in Table 4. At pH 2, 540 µm D_gw_ diet increased SolDM compared with the two largest particle sizes, as did xylanase use compared to the controls (0.144 g vs. 0.126 g, *p* ≤ 0.0001). An interaction between particle size and xylanase inclusion was observed at pH 5 for SolDM (*p* = 0.002), whereby an increment in SolDM was observed with the 660, 1390, and 1700 µm D_gw_ corn diets when xylanase was added but not in the 540 µm D_gw_ diet. Water retention capacity was greater with the 1390 and 1700 µm diets compared with the 660 and 540 µm D_gw_ diets at pH 2, but no effects were noted at pH 5 or with xylanase supplementation.

### 3.3. Growth Performance

**Figure 3:**
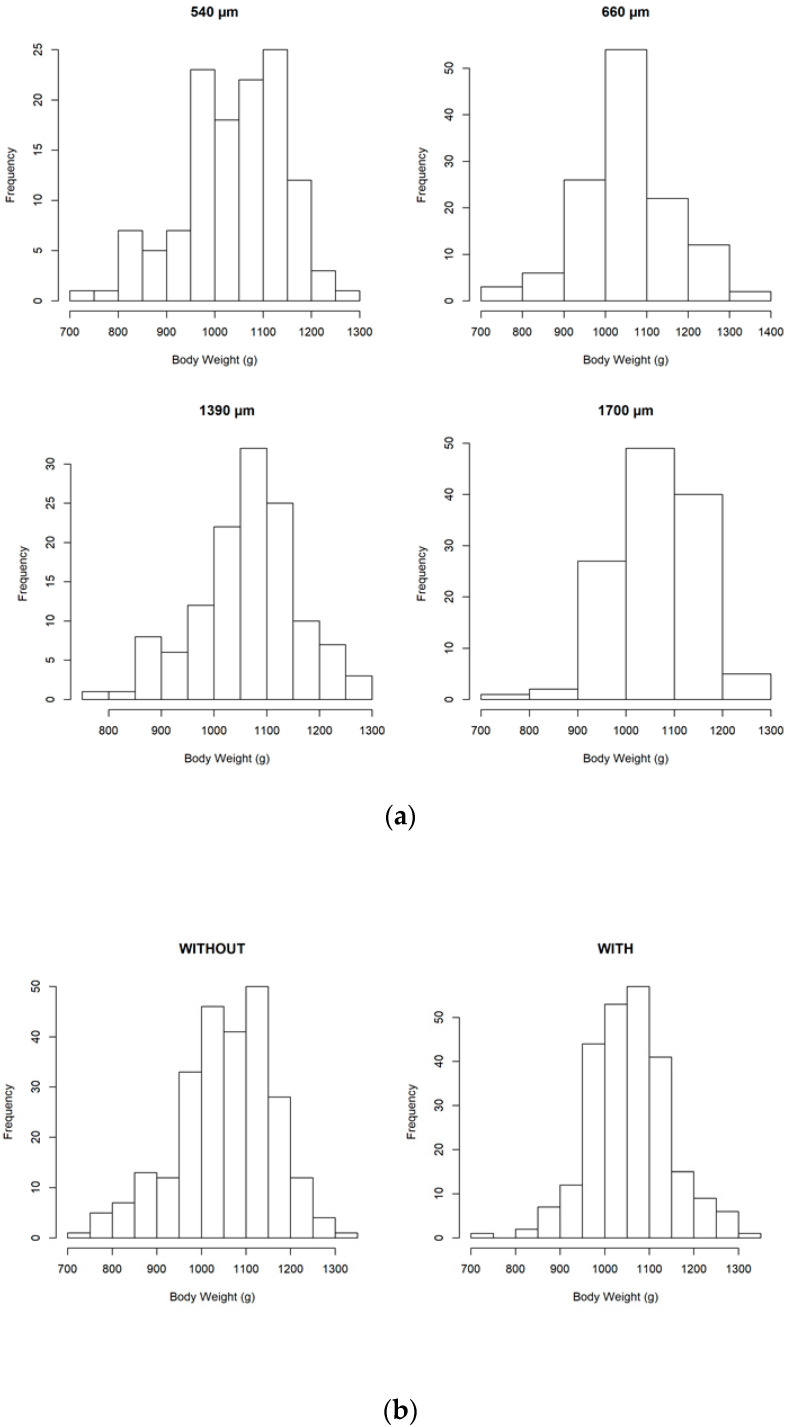
Effect of particle size (**a**) and xylanase inclusion (**b**) on the distribution of body weight (BW).

The effect of particle size and xylanase inclusion on BW and growth performance is described in Figure 3 and summarized in Table 5. Overall broilers mortality was 2.15% (data not shown), and no differences were observed between the experimental treatments (*p* = 0.576). No interactions (*p* > 0.05) were observed between particle size and xylanase supplementation on broiler performance. A statistical trend (*p* = 0.083) was observed for average daily feed intake (ADFI), showing a decrease in the 540 µm D_gw_ corn diet compared to the 1390 µm diet (55.7 vs. 57.3, g/bird/d). The BW CV was significantly affected by both particle size and xylanase. Birds fed the 1390 µm and 1700 µm D_gw_ corn diets had inferior CV compared to those on the 540 and 660 µm D_gw_ corn diets (8.3% & 7.4% vs. 9.9% & 9.2%; *p* = 0.044). Xylanase decreased BW CV (7.7%) compared to the birds fed control diets (9.7%; *p* = 0.003).

### 3.4. Apparent Ileal and Total Tract Digestibility

No interactions in ileal and total tract (Table 6 and Table 7) digestibility were observed between particle size and xylanase. Ileal digestibility was increased when feeding the 1390 and 1700 µm compared with the 540 and 690 µm D_gw_ corn diets for protein (86.2% & 86.2% vs. 83.8% & 84.4%; *p* = 0.001) and apparent energy digestibility (78.1% & 78.8% vs. 76.6% & 76.4%; *p* = 0.029). Apparent ileal digestibility of energy (*p* = 0.029) and OM digestibility (*p* = 0.007) were found to be decreased in birds fed the xylanase diet. Total tract digestibility showed that birds fed 540 µm D_gw_ corn diet had inferior protein digestibility compared to birds fed the 1390 and 1700 µm D_gw_ corn diets (66.8% vs. 70.2% & 70.1%; *p* = 0.001). Apparent total tract energy and DM digestibility were both reduced (*p* = 0.025 and *p* = 0.001, respectively) with xylanase supplementation.

The estimation of total intake of digestible nutrients showed a significant increment in the ileal samples on protein and energy for coarse particle diets (1700 and 1390 µm D_gw_ corn diets) compared to the fine diets (540 and 660 µm D_gw_ corn diets). Additionally, total intake of digestible nutrients showed a significant increment in the fecal samples on protein, energy, and OM for 1700, 1390, and 660 µm D_gw_ corn diets compared to the 540 µm D_gw_ corn diet.

### 3.5. Relative Organ Weights

There were no interactions between particle size and xylanase inclusion for relative organ weights, and the relative weights of duodenum, jejunum, ileum, and small intestine were not influenced by the main factors (Table 8). Coarse diets (1390 and 1700 µm D_gw_) resulted in heavier (*p* < 0.001) gizzards compared with the 540 µm D_gw_ corn diet (1.78 & 1.91 vs. 1.54 g/100 g BW), whilst the caecum was heavier (*p* = 0.004) when birds were fed the 540 µm D_gw_ corn diet compared with the 1390 µm D_gw_ corn diet (0.42 vs. 0.38 g/100 g BW).

### 3.6. Microbial Diversity Analysis

**Figure 4:**
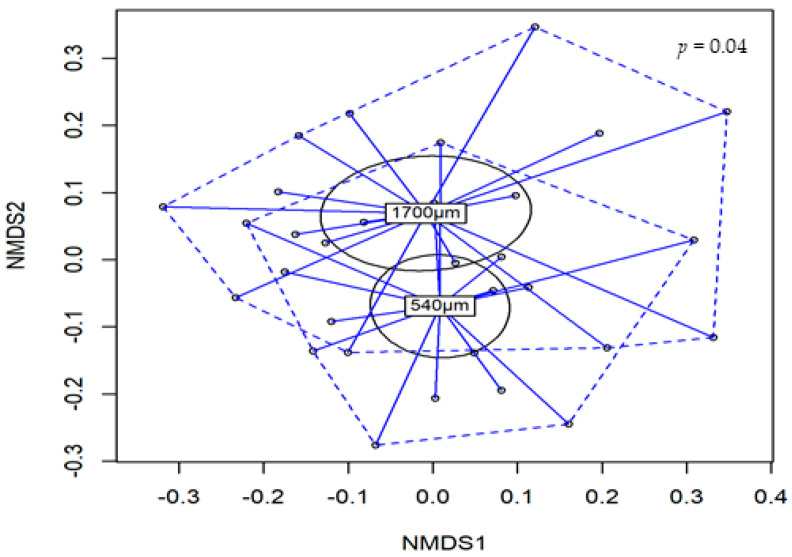
Comparison of beta-diversity of caecum microbiota between 540 and 1700 µm D_gw_ corn diets. The nonmetric-multidimensional scaling (NMDS) plots was generated using Bray-Curtis distances. *p*-Value was obtained from adonis analysis.

No interactions were observed in the analysis of alpha and beta diversity. Three alpha-diversity estimators were performed (Simpson, Shannon and Inverse Simpson). Birds fed the 1700 µm D_gw_ corn diet showed greater diversity than birds fed the 540 µm D_gw_ (*p* < 0.05; Table 9) corn diet in Shannon and Inverse Simpson estimators. There were no effects of xylanase supplementation (*p* > 0.10) on any estimate of diversity. For the beta-diversity, 1700 µm D_gw_ corn diet had a different (*p* = 0.04) diversity composition compared to the 540 µm D_gw_ corn diet (Figure 4).

### 3.7. Composition of Gut Microbiota

**Figure 5:**
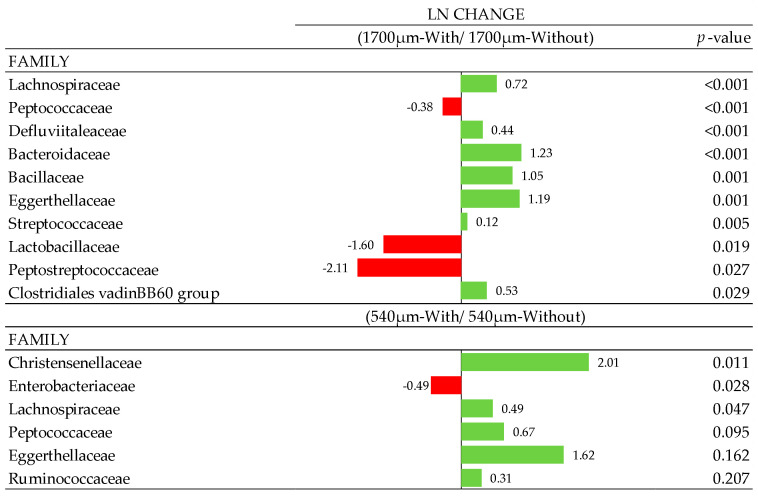
Ln changes promoted by xylanase inclusion in 540 and 1700 µm D_gw_ corn diets (fold discovery rate *p*-adjusted < 0.20) in taxa. Positive values (▮) and negative values (▮) indicate greater and lower abundance. Taxa are sorted by level of significance (from higher to lower). Differences presented are based on all taxa detected in samples per diet.

**Figure 6:**
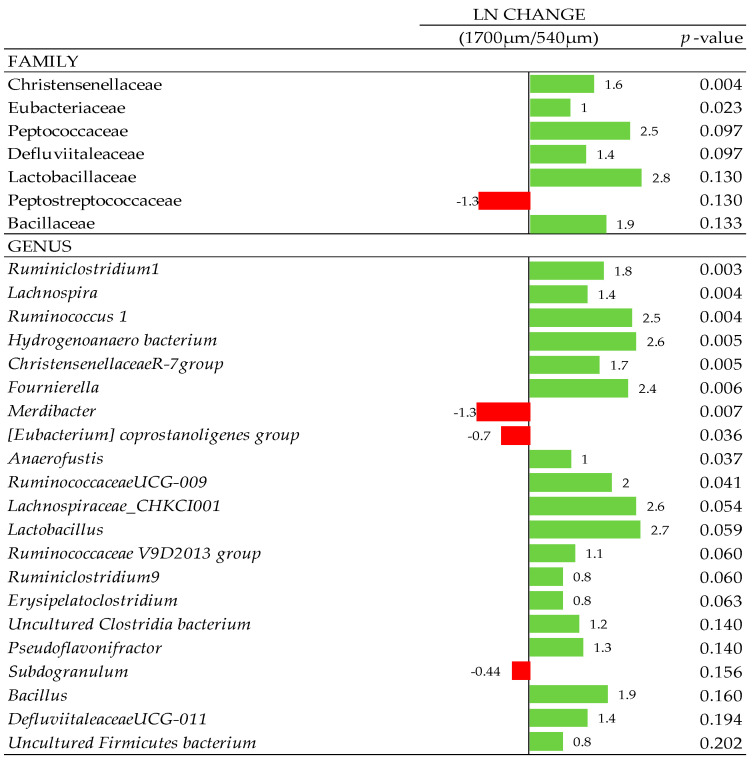
Ln changes promoted by the particle size (1700 and 540, µm) of D_gw_ corn diet (fold discovery rate *p*-adjusted < 0.20) in taxa. Positive values (▮) and negative values (▮) indicate greater and lower abundance. Taxa are sorted by level of significance (from higher to lower). Differences presented are based on all taxa detected in samples per diet.

**Figure 7:**
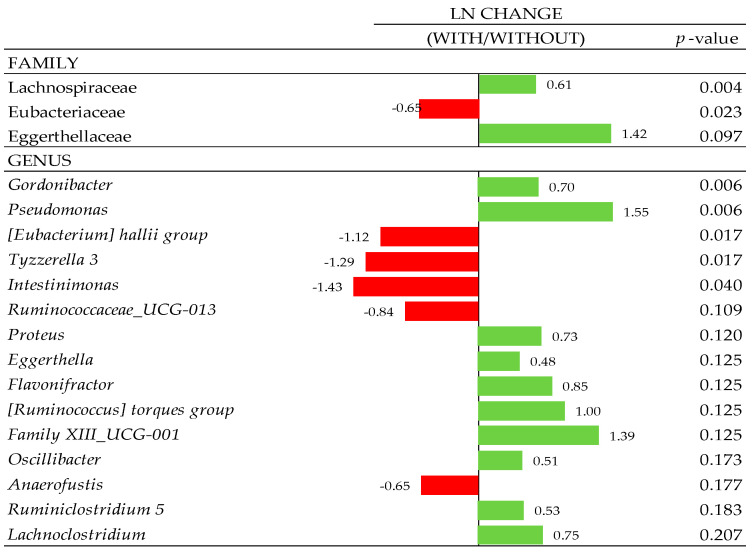
Ln changes promoted by xylanase inclusion (fold discovery rate *p*-adjusted < 0.20) in taxa. Positive values (▮) and negative values (▮) indicate greater and lower abundance. Taxa are sorted by level of significance (from higher to lower). Differences presented are based on all taxa detected in samples per diet.

At the phylum level, 7 phyla were determined, with Firmicutes predominant with a total abundance >83%, followed by Bacteroidetes, Tenericutes, Proteobacteria, and Actinobacteria and other less abundant phyla. No effects were observed at phylum, class, and order levels (*p* < 0.10; data not shown). Some family members were influenced with xylanase supplementation in the 540 and 1700 µm D_gw_ corn diets (Figure 5). Defluviitaleaceae, Bacteroidaceae, Bacillaceae, Eggerthellaceae, Streptococcaceae, Lachnospiraceae, and Clostridiales vadinBB60 group were increased (*p* < 0.05) in the 1700 µm diet when the xylanase was added. On the other hand, Peptococcaceae, Peptostreptococcaceae and Lactobacillaceae families were reduced when the enzyme was supplemented in this diet. In the 540 µm D_gw_ corn diet, Christensenellaceae, Lachnospiraceae, and Peptococcaceae (*p* < 0.10) were increased when xylanase was added. In contrast, the relative abundance of Enterobacteriaceae was reduced in xylanase supplemented birds (*p* < 0.05). Additionally, particle size and xylanase inclusion as a main effect showed significant changes (Figure 6 and Figure 7) at family (f.) and genus (g.) levels, individually (Figure 6 and Figure 7). Therefore, f. Christensenellaceae, f. Eubacteriaceae, g. *Ruminiclostridium* 1, g. *Lachnospira*, g. *Ruminococcus* 1, g. *Hydrogenoanaerobacterium*, g. *Christensenellaceae R-7 group*, g. *Fournierella*, g. *Merdibacter*, g. *Eubacterium coprostanoligenes group*, g. *Anaerofustis* and g. *Ruminococcaceae UCG-009* showed a greater presence in birds fed with the 1700 µm D_gw_ corn diet compared to birds fed the 540 µm D_gw_ corn diet (*p*-adjust ≤ 0.05). Other changes at the family level were observed due to particle size such as the increase in 1700 µm D_gw_ corn diet of Christensenellaceae, Eubacteriaceae, Peptococcaceae, Defluviitaleaceae, Lactobacillaceae, and Bacillaceae, and a reduction of Peptostreptococcaceae in 1700 µm D_gw_ corn diet compared to the 540 µm D_gw_ corn diet (*p* < 0.15). Xylanase inclusion showed a significant increment in f. Lachnospiraceae, g. *Gordonibacter,* and g. *Pseudomonas*, and a reduction of f. Eubacteriaceae, g. *Eubacterium hallii group*, g. *Tyzzerella 3,* and g. *Intestinimonas* (*p* < 0.05).

### 3.8. Determination of Short-Chain Fatty Acids

For SCFA, a significant interaction between particle size and xylanase inclusion was observed for cecal digesta propionic acid (*p* = 0.027; Table 10). Xylanase supplementation in the 1700 µm D_gw_ corn diet reduced propionic acid concentration compared to the same diet without the enzyme (3.8% vs. 5.0%), but this effect was not observed in the other particle size diets. Lactic acid concentration was increased in birds fed 540 µm D_gw_ corn diet than those fed the coarse 1700 µm D_gw_ corn diet (9.4% vs. 7.3%; *p* = 0.025) and total volatile fatty acid (VFA) was increased with the 1700 µm D_gw_ corn diet compared to the 540 µm D_gw_ corn diet (92.8% vs. 90.6%; *p* = 0.029).

## 4. Discussion

The broiler chickens in the current study performed above breed standards (959 g vs. 1055 g at day 21). This result may reflect optimum management and environmental conditions produced by the cage allocation and feeding with well-equilibrated corn soybean diets. This sets the experimental results in context.

### 4.1. Particle Size

In the present study, the effect of the second grinding of pelletizing clearly reduces the particle size of the coarse and medium diets, narrowing the gap with the fine diets. Thus, the differences in particle size distribution post-pelletizing, added to the good management and environmental conditions, were not sufficiently large to cause any differences in bird performance. However, significant changes were observed due to the particle size on the in-vitro SolDM and WRC, CV of body weight, nutrient digestibility, organ relative weight and changes in the microbiota profile. Thus, the 1700 um Dgw corn diet was associated with heavier relative gizzard weights, greater WRC and ileal digestibility of protein, energy, DM and OM, and greater alpha and beta microbiota diversity with many changes in the relative abundance of taxonomic bacteria composition. The benefits of gizzard development are described in the literature and include a prevention of pathogenic bacteria entering the small intestine [32,33,34], increased gizzard contractions, HCl production and gastrointestinal reflux [33,35,36]. Moreover, the increase of HCl secretion and exposure time of nutrients to digestive enzymes may improve nutrient digestibility [37,38] and enhance GIT motility [33,35,36]. Lastly, it has been reported that a lower pH of gizzard contents may increase pepsin activity [35] and improve protein digestion, confirming the important influence coarse particles have in poultry gut function. In this way, several studies in poultry with different cereals showed an improvement in the size of the gizzard and the nutrient utilization when coarse diets or post-pelleting of whole grain were used [6,7,18]. Therefore, Sing and Ravindran [39] indicated that 115 g/kg of ground corn can be replaced by whole corn in broiler starter diets without adverse effects on growth performance; however, the increase in the relative weight of the gizzard at 21 days was lower than in the present study (15,4 vs. 19.1, g/kg, respectively). Alternatively, Vukmirovic et al. [9] reported that the use of hammer mill with a sieve of 9 mm and a roller pellet press of 2 mm was the best option to conserve coarse particles with higher PDI and reduce the cost of pelletizing. In the present study, a similar configuration was used to obtain the coarsest corn treatments (1390 and 1700 µm) with similar results preserving coarse particles and PDI.

On the other hand, although SolDM at pH 5 was increased in the 540 µm D_gw_ corn diet, no positive changes were observed in the nutrient digestibility. Contrary, the apparent ileal digestibility of protein and energy were significantly decreased in this diet. Thus, the negative effect on the gizzard development, added to the decrease in PDI, could explain the inferior digestibility and increased CV observed in this treatment group. Thus, a positive correlation between pellet durability and feed efficiency has been described [40].

As we noticed previously, important changes in microbiota composition, such as the greater alpha and beta diversity, were observed when birds were fed with 1700 µm D_gw_ corn diet. This suggests that intestinal microbiota diversity could be improved when the proximal digestive tract is developed with coarse particles. Moreover, coarse particles increased the relative abundance of *Ruminoclostridium1*, *Lachnospira and Ruminococcus1*, and numerically increased families like Lactobacillaceae and Bacillaceae, suggesting a greater relative abundance of butyric, propionic, and acetic producers [41,42,43]. Some of these bacteria are related to a better performance in broilers [44,45]. Thus, it seems that an improvement in the development of proximal GIT due to feeding coarser particle pelleted diets promoted positive changes in the intestinal microbiome, with significant increases in nutrient digestibility and VFA production. Although no significant effects were observed on performance, the increased BW homogeneity observed when birds were fed the 1700 µm D_gw_ corn diet may suggest a better GIT development with positive effects on gut functions.

### 4.2. Xylanase

In-vitro results showed that xylanase increased the DM solubility at pH 2, and interacted with particle size at pH 5, showing a greater solubility in coarse diets. These results suggest a release of encapsulated nutrients or/and the production of XOS or AXOS from the plant cell walls, as suggested by some authors [46,47,48]. Thus, several studies have reported benefits when XOS were included in pig and poultry diets [49,50,51,52]. Therefore, the XOS obtained when xylanase was added could improve the relative abundance of Lachnospiraceae family, and several members of this family are recognized as butyric-producer bacteria [51]. Consequently, the significant increment of these families suggest that these specific bacterial communities, fiber degrading bacteria, could improve the production of SCFA that are correlated with gut health improvements, but are also a good source of energy for the intestine [53]. On the contrary, the current study did not show differences in SCFA when xylanase was added, which might be related with the extremely fast turnover rate of SCFA from the intestine into the blood [54], and/or the preference for this VFA over others in the intestinal epithelial cells and the liver [55]. Thus, the GIT is not only a site for digestion and absorption of nutrients but also experiences enormous interactions with the microbiota, and functions as a metabolic and immunological organ [56]. Thus, xylanase supplementation could have a role in reducing the immunological stress in the gut, probably through microbiota modulation and subsequent reduction in the metabolic yield of an innate immune system, which increases the availability of nutrients for growth rather than maintenance. In the present study, although there were no changes in performance, xylanase improved BW homogeneity, which is intriguing, given the digestibility data. Likewise, predicting long-term effects of a diet on performance parameters, such as BW gain or FCR, based on nutrient digestibility responses to xylanase also appeared to be incongruous in previous studies [49]. However, when estimating the total intake of digestible nutrients, using digestibility coefficients, the low digestibility associated with the xylanase supplementation disappears, showing a better adjusted estimation on digestibility effects.

### 4.3. Particle Size and Xylanase Interaction

No interaction between particle size and xylanase supplementation was observed for any productive parameters in the present study. In general, the agreement is that smaller particles increase the specific surface of feed particles allowing better contact with the enzymes. However, significant benefits on performance and digestibility have been reported when exogenous enzymes supplementation was accompanied by coarse particles or whole grain in the diets [4,18]. Therefore, it is reasonable to suggest that the increasing gizzard size improves exogenous enzyme response due to increased peristalsis, mixing, pH, and gut stimulation. On the latter, Kheravii et al. [57] demonstrated that the coarse particles modulate expression of genes encoding important digestive enzymes and nutrient transporters, with consequent benefits in the performance of birds [57]. Although, no significant interactions on digestibility or performance were observed in the present study, there were significant changes in the microbiota profile of both 1700 and 540 µm D_gw_ corn diets when xylanase was added. Xylanase addition supported the growth of bacteria producing SCFA from polysaccharides, such as the Defluviitaleaceae, Bacillaceae, and Lachnospiraceae families [58,59] in the 1700 µm D_gw_ corn diet, and Christensenellaceae and Lachnospiraceae families [60,61] in the 540 µm D_gw_ corn diet. Specifically, the Lachnospiraceae family, described as butyric acid producers, have genes coding for enzymes (xylanases, cellulases) to degrade a wide variety of polysaccharides [62], and as a result, may have protective properties against digestive disorders in pigs [63]. It is important to highlight that the Enterobacteriaceae family was reduced when xylanase was included in 540 µm D_gw_ corn diet, which could have a role in explaining the reduction in the BW CV observed with this diet. Furthermore, inhibitory effects of SCFA on pathogenic bacteria, such as *Salmonella*, a zoonotic agent belonging to the Enterobacteriaceae family, have also been described [64,65]. Therefore, the promotion of specific bacterial communities that can degrade complex substrates, such as non-starch polysaccharides, leading to better growth performance of the broiler chicken, is certainly the most important mechanism that is discussed when xylanase is proposed as a feed additive in corn based diets for poultry.

Overall, particle size determined the development and functionality of the GIT, which instead, clearly influence the gut microbiota, showing different responses to the dietary supplementation of xylanase.

## 5. Conclusions

It is concluded that xylanase supplementation and particle size of corn interact in the intestinal environment, producing changes in microbial composition. Coarse diets and xylanase supplementation improved body weight homogeneity, gut development, and microbiota modulation.

## Figures and Tables

**Table 1:** Ingredient and calculated composition of the d 0 to 21 experimental diets ^1^.

Items	Geometric Mean of Corn (µm)			
	540	660	1390	1700
**Ingredients, %**				
Corn	53.4			
Soybean meal 47	38.4			
Palm oil	2.50			
Soy oil	2.69			
Salt	0.31			
DL-Methionine	0.29			
Lysine HCl	0.14			
Threonine	0.03			
Limestone	0.97			
Mono dicalcium phosphate	0.94			
Vitamin premix ^2^	0.40			
**Calculated composition (%, as feed)**				
Apparent Metabolizable Energy, kcal/kg	2900			
Crude protein	22.3			
Calcium	0.90			
Available P	0.45			
Crude fat	7.68			
D Me + Cys	0.98			
D Lys	1.18			
**Analyzed composition ^3^, %**				
Gross energy, kcal/kg	4264			
Dry matter	90.3			
Crude protein	22.3			
Starch	27.7			
Ash	5.6			

^1^ Hydrochloric acid (HCl), phosphorus (P), methionine (Me), cysteine (Cys), lysine (Lys). ^2^ Provided per kg of feed: Vitamin A (retinil acetate) 10,000 UI; Vitamin D (Vitamin D_3_) (Colecalciferol) 4800 UI; Vitamin E/acetate de tot-rac-3- tocopheril) 45 mg; Vitamin K_3_ (MNB Menadiona nicotinamida bisulfit) 3 mg; Vitamin B_1_ (Tiamin mononitrat) 3 mg; Vitamin B2 (Riboflavin) 9 mg; Vitamin B6 (Piridoxin Chlorhidrate) 4.5 mg; Vitamin B12 (cyanocobalamine) 0.04 mg; Nicotinamida 51 mg; Pantotenic Acid (Calcium D-pantotenate) 16.5 mg; Biotin (D-(+)-biotin) 0.15 mg; Folic Acid 1.8 mg; Choline chloride 350 mg; Iron (Iron sulphate monohydrate) 54 mg; Zinc (Zn, zinc oxide) 66 mg; Manganes (Mn, Manganes oxide) 90 mg; Iodine (I, Calcium Iondine Anhydrate) 1.2 mg; Selenium (Se, Sodium Selenate) 0.18 mg; Copper (Cu, copper Sulphate Penthahydrate) 12 mg; Etoxiquin 4 mg; D,L-Malic acid 60 mg; Fumaric acid 75 mg; Sepiolite 907 mg; Vermiculite 2001 mg; Colloidal silica 45 mg. ^3^ The range of analyzed nutrients was; gross energy from 4259 to 4269 kcal/kg, dry matter from 90.1 to 90.6%, crude protein from 21.3 to 22.5, ash from 5.4 to 5.7% and starch from 25.7 to 28.6 % (as is).

**Table 2:** Analyzed enzyme activities of xylanase in diets (d 0 to 21).

Diets ^1^	Xylanase ^2^ (BXU/kg)
540	<2000
660	<2000
1390	<2000
1700	<2000
540 + Xylanase	12,400
660 + Xylanase	13,900
1390 + Xylanase	12,700
1700 + Xylanase	14,200

^1^ Diets consisted of a four geometric mean diameter (D_gw_) for corn (540, 660, 1390, and 1700, µm), and two levels of xylanase supplementation (0 or 16,000 BXU/kg diet). ^2^ One BXU is defined as the amount of enzyme that produces one nmol reducing sugars from birchwood xylan in ones at 50 °C and pH 5.3.

**Table 3:** Geometric mean diameter (D_gw)_ and standard deviation (S_gw_) of corn, mash, and pellet feed ^1^.

D_gw_ (µm)			S_gw_ (µm)		
Corn	Mash Diet	Pellet Diet	Corn	Mash Diet	Pellet Diet
540	617	488	2.33	2.79	2.03
660	701	549	2.37	2.85	2.13
1390	1032	583	2.54	3.01	2.11
1700	1170	637	2.48	3.04	2.32

^1^ The four D_gw_ particle sizes (540, 660, 1390, and 1700, µm) of corn corresponding to 1500, 1200, 600, and 450 rpm mill speed, and 5, 5, 8, and 8 mm mill screen size, respectively. The soybean meal for all diets was milled at 1500 rpm and 5 mm screen size for all diets.

**Table 4:** Effects of particle size and xylanase inclusion on the in-vitro analysis ^1,2^.

		pH 2		pH 5	
Particle Size	Xylanase	SolDM	WRC	SolDM	WRC
540	-	0.136	2.16	0.149 ^a^	2.25
	+	0.146	2.21	0.157 ^a^	2.27
660	-	0.126	2.22	0.135 ^b^	2.28
	+	0.143	2.11	0.153 ^a^	2.23
1390	-	0.122	2.23	0.133 ^b^	2.37
	+	0.141	2.27	0.156 ^a^	2.31
1700	-	0.120	2.26	0.122 ^b^	2.35
	+	0.147	2.28	0.154 ^a^	2.24
SEM ^3^		0.0033	0.044	0.0031	0.058
Main effect					
Particle size	540	0.144 ^a^	2.18 ^b^	0.153	2.27
	660	0.135 ^ab^	2.17 ^b^	0.144	2.26
	1390	0.132 ^b^	2.25 ^a^	0.144	2.34
	1700	0.133 ^b^	2.27 ^a^	0.138	2.30
SEM		0.0021	0.031	0.0002	0.043
Xylanase	-	0.126 ^b^	2.22	0.135	2.32
	+	0.144 ^a^	2.22	0.155	2.28
SEM		0.0013	0.018	0.0014	0.030
*p*-value ^4^					
Particle Size		0.013	0.015	<0.0001	0.443
Xylanase		<0.0001	0.993	<0.0001	0.164
Particle size x Xylanase		0.051	0.105	0.002	0.880

^1^ Data are mean of 5 replicates for each treatment. ^2^ Dry matter solubility (SolDM) and water retention capacity (WRC) at pH 2 and pH 5 ^3^ Standard error of the mean. ^4ab^ Values in the same column with different letters are statistically different (*p* < 0.05).

**Table 5:** Effects on performance ^1^ of particle size and xylanase inclusion ^2^.

Particle Size	Xylanase	BW (g)	ADG (g/d/bird)	ADFI (g/d/bird)	FCR (g/g)	CV (%)
540	-	1032	47.5	55.4	1.17	12.1
	+	1048	48.0	56.1	1.18	7.6
660	-	1050	48.0	56.6	1.18	10.4
	+	1059	48.8	56.9	1.17	8.1
1390	-	1076	49.5	58.2	1.18	8.1
	+	1053	48.2	57.5	1.19	8.5
1700	-	1060	48.6	56.9	1.17	8.1
	+	1066	48.6	57.8	1.18	6.6
SEM ^3^		14.0	0.69	0.84	0.014	0.89
Main effect						
Particle size	540	1040	47.4	55.7	1.18	9.9 ^b^
	660	1055	48.4	56.8	1.17	9.2 ^b^
	1390	1064	48.8	57.9	1.18	8.3 ^a^
	1700	1063	48.6	57.3	1.17	7.4 ^a^
SEM		9.9	0.48	0.59	0.010	0.63
Xylanase	-	1055	48.4	56.8	1.17	9.7 ^b^
SEM	+	1057	48.3	57.1	1.18	7.7 ^a^
		7.0	0.34	0.42	0.007	0.44
*p*-value ^4^						
Particle Size		0.280	0.189	0.083	0.886	0.044
Xylanase		0.836	0.848	0.637	0.486	0.003
Particle size x Xylanase		0.520	0.537	0.762	0.835	0.074

^1^ Body weight (BW), average daily gain (ADG), average daily feed intake (ADFI), 0–21 day feed conversion ratio (FCR) and BW coefficient of variation (CV). ^2^ Data are means of 8 pens with 8 birds (d 0–21) by treatment. ^3^ Standard error of the mean. ^4ab^ Values in the same column with different letters are statistically different (*p* < 0.05).

**Table 6:** Effects of particle size and xylanase inclusion on 21-day apparent ileal digestibility and intake of digestible nutrients ^1,2^.

Main Effects		Ileal Digestibility (%)				Intake of Digestible Nutrients			
		Protein	Energy	OM	Starch	Protein g	Energy kcal	OM g	Starch G
Particle size	540	83.8 ^b^	76.6 ^b^	74.1	96.8	220 ^b^	3848 ^b^	776 ^b^	319
	660	84.4 ^b^	76.4 ^b^	73.5	96.1	221 ^b^	3839 ^b^	770 ^b^	320
	1390	86.2 ^a^	78.1 ^a^	75.1	96.5	235 ^a^	4045 ^a^	811 ^a^	328
	1700	86.2 ^a^	78.8 ^a^	76.2	96.5	233 ^a^	4033 ^a^	810 ^a^	326
SEM		0.47	0.66	0.78	0.31	3.7	53.1	10.9	3.6
Xylanase	-	85.4	78.2 ^a^	75.8 ^a^	96.7	225	3968	797	322
	+	84.9	76.8 ^b^	73.7 ^b^	96.3	229	3914	786	324
SEM^2^		0.35	0.47	0.55	0.22	2.9	36.6	7.9	2.5
*p*-value ^3^									
Particle Size		0.001	0.029	0.086	0.525	0.011	0.003	0.013	0.135
Xylanase		0.29	0.037	0.007	0.139	0.429	0.277	0.311	0.605
Particle size x Xylanase		0.515	0.775	0.867	0.099	0.537	0.645	0.486	0.511

^1^ Data are a pool of 3 birds ileal contend per pen with 8 pen per treatment. ^2^ Protein, energy, dry matter (DM), organic matter (OM) and starch. ^2^ Standard error of the mean. ^3ab^ Values in the same column with different letters are statistically different (*p* < 0.05).

**Table 7:** Effects of particle size and xylanase inclusion on 21-day apparent total tract digestibility and intake of digestible nutrients ^1,2^.

Main Effects		Total Tract Digestibility (%)			Intake of Digestible Nutrients		
		Protein	Energy	OM	Protein g	Energy kcal	OM g
Particle size	540	66.8 ^b^	77.6	74.6	171 ^c^	3862 ^b^	794 ^b^
	660	69.5 ^ab^	78.5	74.9	182 ^b^	4016 ^ab^	822 ^a^
	1390	70.2 ^a^	78.1	74.9	189 ^a^	4066 ^a^	836 ^a^
	1700	70.1 ^a^	78.7	74.9	185 ^ab^	4128 ^a^	827
SEM		0.61	0.44	0.55	2.2	64.1	9.4
Xylanase	-	69.5	78.7 ^a^	75.3	181	3987	818
	+	68.9	77.7 ^b^	74.3	183	4050	821
SEM ^3^		0.41	0.3	0.37	1.5	42.6	6.5
*p*-value ^4^							
Particle Size		0.001	0.296	0.961	<0.0001	0.031	0.016
Xylanase		0.367	0.025	0.058	0.434	0.317	0.700
Particle size x Xylanase		0.788	0.633	0.765	0.857	0.293	0.485

^1^ Data are a pool of 8 birds feces samples per pen with 8 pen per treatment. ^2^ Protein, energy, dry matter (DM) and organic matter (OM) ^3^ Standard error of the mean. ^4abc^ Values in the same column with different letters are statistically different (*p* < 0.05).

**Table 8:** Effects of particle size and xylanase inclusion on 21-day relative organs weight ^1^.

Main Effects		Gizzard	Duodenum	Jejunum	Ileum	Ceca	Small Intestine ^2^
		g/100 g of Body Weight					
Particle size	540	1.54 ^c^	1.11	1.41	1.29	0.42 ^a^	3.82
	660	1.66 ^bc^	1.14	1.38	1.28	0.40 ^ab^	3.83
	1390	1.78 ^ab^	1.15	1.36	1.22	0.38 ^b^	3.75
	1700	1.91 ^a^	1.13	1.27	1.29	0.40 ^ab^	3.71
SEM ^3^		0.051	0.033	0.042	0.032	0.011	0.089
Xylanase	-	1.75	1.15	1.35	1.28	0.39	3.80
SEM	+	1.69	1.11	1.37	1.25	0.39	3.76
		0.035	0.023	0.029	0.024	0.007	0.063
*p*-value ^4^							
Particle Size		<0.0001	0.826	0.102	0.353	0.004	0.767
Xylanase		0.288	0.227	0.599	0.247	0.831	0.407
Particle size x Xylanase		0.721	0.646	0.805	0.353	0.314	0.917

^1^ Data are means of 3 birds per cage with 8 pens per treatment. ^2^ Small intestine = Duodenum + Jejunum + Ileum. ^3^ Standard error of the mean. ^4abc^ Values in the same column with different letters are statistically different (*p* < 0.05).

**Table 9:** Effects particle size ^1^ and xylanase inclusion in alpha diversity on 21-day cecal microbiota ^2^.

Main Effects		Shannon	Simpson	Inverse-Simpson
Particle size	540	4.22 ^b^	0.96	31.0 ^b^
	1700	4.44 ^a^	0.97	44.1 ^a^
SEM ^3^		0.068	0.004	4.49
Xylanase	-	4.28	0.96	35.4
	+	4.38	0.96	39.4
SEM		0.068	0.004	4.49
*p*-value ^4^				
Particle size		0.034	0.146	0.048
Xylanase		0.282	0.471	0.566
Particle size x Xylanase		0.092	0.155	0.290

^1^ Only 1700 and 540 µm D_gw_ diets, with and without xylanase, were analyzed. ^2^ Data are a mean of 3 birds per pen with 8 pen per treatment. ^3^ Standard error of the mean. ^4ab^ Values in the same column with different letters are statistically different (*p* < 0.05).

**Table 10:** Effect of particle size ^1^ and xylanase inclusion on total short-chain fatty acid ^2,3^.

					Acid				
		SCFA	VFA	BCFA	Acetic	Propionic	Butyric	Valeric	Lactic
Particle Size	Xylanase	mM	%						
540	-	97.3	90.5	1.93	71.3	4.1 ^bc^	12.1	1.07	9.5
	+	98.5	90.7	1.80	71.4	4.7 ^abc^	11.7	0.97	9.3
660	-	96.9	91.5	1.73	72.3	4.4 ^abc^	12.0	0.95	8.5
	+	93.0	92.1	1.95	71.5	5.3 ^a^	12.3	1.01	7.8
1700	-	93.1	92.5	1.85	71.7	5.0 ^ab^	12.9	1.03	7.5
	+	91.8	92.9	1.81	74.7	3.8 ^c^	11.7	0.83	7.0
SEM ^5^		4.33	0.802	0.159	1.43	0.40	0.88	0.090	0.80
Main effect									
Particle size	540	97.9	90.6 ^b^	1.86	71.3	4.5	11.9	1.03	9.4 ^a^
	660	95.0	91.8 ^ab^	1.84	71.9	4.8	12.2	0.98	8.1 ^ab^
	1700	92.5	92.8 ^a^	1.83	73.2	4.4	12.3	0.94	7.3 ^b^
SEM		3.06	0.56	0.112	1.01	0.28	0.62	0.063	0.56
Xylanase	-	95.8	87.7	1.83	71.8	4.5	12.4	1.02	8.48
	+	94.4	86.9	1.85	72.5	4.6	11.9	0.94	8.03
SEM^4^		2.50	2.52	0.092	0.82	0.23	0.51	0.052	0.462
*p*-value ^5^									
Particle Size		0.459	0.039	0.981	0.416	0.519	0.911	0.628	0.039
Xylanase		0.711	0.565	0.898	0.527	0.697	0.552	0.288	0.507
Particle size*Xylanase		0.836	0.947	0.552	0.403	0.028	0.721	0.349	0.954

^1^ Only 540, 660 and 1700 µm D_gw_ corn diets, with and without the enzyme were analyzed. ^2^ Data are a mean of 5 birds per pen with 8 pen per treatment. ^3^ Volatile fatty acid (VFA), branched-chain fatty acid (BCFA) and lactic acid concentrations in ceca content are expressed in percentage of the total SCFA. ^4^ Standard error of the mean. ^5abc^ Values in the same column with different letters are statistically different (*p* < 0.05).

## References

[1] Vukmirović Đ., Čolović R., Rakita S., Brlek T., Đuragić O., Solà-Oriol D. (2017). Importance of feed structure (particle size) and feed form (mash vs. pellets) in pig nutrition–A review. Anim. Feed Sci. Technol..

[2] Kersten J., Rohde H.R., Nef E. (2005). Principles of Mixed Feed Production.

[3] Roche M. (1981). Comportement alimentaire et motricité digestive des oiseaux. Reprod. Nutr. Dev..

[4] Córdova-Noboa H.A., Oviedo-Rondón E.O., Ortiz A., Matta Y., Hoyos S., Buitrago G.D., Martinez J.D., Yanquen J., Peñuela L., Sorbara J.O.B. (2020). Corn drying temperature, particle size, and amylase supplementation influence growth performance, digestive tract development, and nutrient utilization of broilers. Poult. Sci..

[5] Abd El-Wahab A., Kriewitz J.-P., Hankel J., Chuppava B., Ratert C., Taube V., Visscher C., Kamphues J. (2020). The Effects of Feed Particle Size and Floor Type on the Growth Performance, GIT Development, and Pododermatitis in Broiler Chickens. Animals.

[6] Perera W.N.U., Abdollahi M.R., Zaefarian F., Wester T.J., Ravindran V. (2020). The interactive influence of barley particle size and enzyme supplementation on growth performance, nutrient utilization, and intestinal morphometry of broiler starters. Poult. Sci..

[7] Mtei A.W., Abdollahi M.R., Schreurs N.M., Ravindran V. (2019). Impact of corn particle size on nutrient digestibility varies depending on bird type. Poult. Sci..

[8] Svihus B., Kløvstad K.H., Perez V., Zimonja O., Sahlström S., Schüller R.B., Jeksrud W.K., Prestløkken E. (2004). Physical and nutritional effects of pelleting of broiler chicken diets made from wheat ground to different coarsenesses by the use of roller mill and hammer mill. Anim. Feed Sci. Technol..

[9] Vukmirović D., Fišteš A., Lević J., Čolović R., Rakić D., Brlek T., Banjac V. (2017). Possibilities for preservation of coarse particles in pelleting process to improve feed quality characteristics. J. Anim. Physiol. Anim. Nutr..

[10] Abdollahi M.R., Ravindran V., Svihus B. (2013). Pelleting of broiler diets: An overview with emphasis on pellet quality and nutritional value. Anim. Feed Sci. Technol..

[11] Choct M., Kocher A., Waters D.L.E., Pettersson D., Ross G. (2004). A comparison of three xylanases on the nutritive value of two wheats for broiler chickens. Br. J. Nutr..

[12] Latham R.E., Williams M.P., Flores C., O’Neill H.V.M., York T.W., Lee J.T. (2016). Impact of variable corn nutrient content, AME prediction, and xylanase inclusion on growth performance. J. Appl. Poult. Res..

[13] Tang D.F., Liu X.X., Shi X.G., Aftab U. (2017). Effect of cereal type and Xylanase supplementation on nutrient retention and growth performance of broilers. J. Appl. Poult. Res..

[14] Bedford M.R., Apajalahti J., Bedford M.R., Gary G. (2000). Microbial interactions in the response to exogenous enzyme utilization. Enzymes in Farm Animal Nutrition.

[15] Bedford M.R., Classen H.L. (1992). Reduction of Intestinal Viscosity through Manipulation of Dietary Rye and Pentosanase Concentration is Effected through Changes in the Carbohydrate Composition of the Intestinal Aqueous Phase and Results in Improved Growth Rate and Food Conversion Efficie. J. Nutr..

[16] Khadem A., Lourenco M., Delezie E., Maertens L., Goderis A., Mombaerts R., Hofte M., Eeckhaut V., Van Immerseel F. (2016). Does release of encapsulated nutrients have an important role in the efficacy of xylanase in broilers?. Poult. Sci..

[17] Amerah A.M., Ravindran V., Lentle R.G., Thomas D.G. (2008). Influence of particle size and xylanase supplementation on the performance, energy utilisation, digestive tract parameters and digesta viscosity of broiler starters. Br. Poult. Sci..

[18] Wu Y.B., Ravindran V. (2004). Influence of whole wheat inclusion and xylanase supplementation on the performance, digestive tract measurements and carcass characteristics of broiler chickens. Anim. Feed Sci. Technol..

[19] The European Parliament and the Council of the European Union (2010). Directive 2010/63/EU of the European Parliament and of the Council of 22 of September 2010 on the Protection of Animals Used for Scientific Purposes. Off. J. Eur. Union..

[20] Benz C., Goodband B. (2005). Procedures for Three-Sieve Particle Analysis.

[21] Lázaro R., Mateos G.G., FEDNA (2018). Necesidades Nutricionales Para Avicultura: Pollos de Carne y Aves de Puesta.

[22] Baker S., Herrman T. (2002). Evaluating Particle Size.

[23] Miladinovic D. (2009). Standard Wet Sieving Analysis Fôrtek.

[24] Horwitz W., Latimer G.W., AOAC (2005). Official Methods of Analysis of AOAC International.

[25] Anguita M., Gasa J., Martín-Orúe S.M., Pérez J.F. (2006). Study of the effect of technological processes on starch hydrolysis, non-starch polysaccharides solubilization and physicochemical properties of different ingredients using a two-step in vitro system. Anim. Feed Sci. Technol..

[26] Holben W.E., Williams P., Gilbert M.A., Saarinen M., Sarkilahti L.K., Apajalahti J.H.A. (2002). Phylogenetic analysis of intestinal microflora indicates a novel Mycoplasma phylotype in farmed and wild salmon. Microb. Ecol..

[27] Wood D.E., Salzberg S.L. (2014). Kraken: Ultrafast metagenomic sequence classification using exact alignments. Genome Biol..

[28] Caporaso J.G., Kuczynski J., Stombaugh J., Bittinger K., Bushman F.D., Costello E.K., Fierer N., Peña A.G., Goodrich J.K., Gordon J.I. (2010). QIIME allows analysis of high-throughput community sequencing data. Nat. Methods.

[29] Wang Q., Garrity G.M., Tiedje J.M., Cole J.R. (2007). Naïve Bayesian Classifier for Rapid Assignment of rRNA Sequences into the New Bacterial Taxonomy. Appl. Environ. Microbiol..

[30] Oksanen J., Blanchet R.K.F.G., Legendre P., Minchin P.R., O’Hara R.B., Simpson G.L., Solymos P., Henry H.M., Stevens H.W. (2019). Package ‘vegan.’ In R Packag. https://cran.r-project.org/web/packages/vegan/vegan.pdf.

[31] Paulson N.J., Olson N.D., Braccia D.J., Wagner J., Talukder H., Pop M., Bravo H.C. (2019). Package ‘metagenomeSeq’. https://www.bioconductor.org/packages/release/bioc/html/metagenomeSeq.html.

[32] Cumming R.B. (1994). Opportunities for whole grain feeding. Proceedings of the 9th European Poultry Conference.

[33] Engberg R.M., Hedemann M.S., Jensen B.B. (2002). The influence of grinding and pelleting of feed on the microbial composition and activity in the digestive tract of broiler chickens. Br. Poult. Sci..

[34] Bjerrum L., Pedersen K., Engberg R.M. (2005). The Influence of Whole Wheat Feeding on Salmonella Infection and Gut Flora Composition in Broilers. Avian Dis..

[35] Gabriel I., Mallet S., Leconte M. (2003). Differences in the digestive tract characteristics of broiler chickens fed on complete pelleted diet or on whole wheat added to pelleted protein concentrate. Br. Poult. Sci..

[36] Amerah A.M., Lentle R.G., Ravindran V. (2007). Influence of Feed Form on Gizzard Morphology and Particle Size Spectra of Duodenal Digesta in Broiler Chickens. J. Poult. Sci..

[37] Nir I., Hillel R., Ptichi I., Shefet G. (1995). Effect of Particle Size on Performance. Grinding Pelleting Interactions. Poult. Sci..

[38] Carré B. (2000). Effets de la taille des particules alimentaires sur les processus digestifs chez les oiseaux d’élevage. Prod. Anim..

[39] Singh Y., Ravindran V. (2019). Influence of feeding whole maize, differing in endosperm hardness, on the performance, nutrient utilisation and digestive tract development of broiler starters. J. Appl. Anim. Nutr..

[40] Carré B., Muley N., Gomez J., Oury F.-X., Laffitte E., Guillou D., Signoret C. (2005). Soft wheat instead of hard wheat in pelleted diets results in high starch digestibility in broiler chickens. Br. Poult. Sci..

[41] Lin H., An Y., Hao F., Wang Y., Tang H. (2016). Correlations of Fecal Metabonomic and Microbiomic Changes Induced by High-fat Diet in the Pre-Obesity State. Sci. Rep..

[42] Vital M., Howe A.C., Tiedje J.M. (2014). Revealing the Bacterial Butyrate Synthesis Pathways by Analyzing (Meta)genomic Data. MBio.

[43] Chai L.-J., Lu Z.-M., Zhang X.-J., Ma J., Xu P.-X., Qian W., Xiao C., Wang S.-T., Shen C.-H., Shi J.-S. (2019). Zooming in on Butyrate-Producing Clostridial Consortia in the Fermented Grains of Baijiu via Gene Sequence-Guided Microbial Isolation. Front. Microbiol..

[44] Hmani H., Daoud L., Jlidi M., Jalleli K., Ali M.B., Brahim A.H., Bargui M., Dammak A., Ali M. (2017). A Bacillus subtilis strain as probiotic in poultry: Selection based on in vitro functional properties and enzymatic potentialities. J. Ind. Microbiol. Biotechnol..

[45] Xu S., Lin Y., Zeng D., Zhou M., Zeng Y., Wang H., Zhou Y., Zhu H., Pan K., Jing B. (2018). Bacillus licheniformis normalize the ileum microbiota of chickens infected with necrotic enteritis. Sci. Rep..

[46] Munyaka P.M., Nandha N.K., Kiarie E., Nyachoti C.M., Khafipour E. (2016). Impact of combined β-glucanase and xylanase enzymes on growth performance, nutrients utilization and gut microbiota in broiler chickens fed corn or wheat-based diets. Poult. Sci..

[47] Bedford M.R., Cowieson A.J. (2012). Exogenous enzymes and their effects on intestinal microbiology. Anim. Feed Sci. Technol..

[48] Meng X., Slominski B.A., Nyachoti C.M., Campbell L.D., Guenter W. (2005). Degradation of cell wall polysaccharides by combinations of carbohydrase enzymes and their effect on nutrient utilization and broiler chicken performance. Poult. Sci..

[49] Craig A.D., Khattak F., Hastie P., Bedford M.R., Olukosi O.A. (2019). Xylanase and xylo- oligosaccharide prebiotic improve the growth performance and concentration of potentially prebiotic oligosaccharides in the ileum of broiler chickens. Br. Poult. Sci..

[50] Sou H., Lu L., Xu G., Xiao L., Chen X., Xia R., Zhang L., Luo X. (2015). Effectiveness of dietary xylo-oligosaccharides for broilers fed a conventional corn-soybean meal diet. J. Integr. Agric..

[51] De Maesschalck C., Eeckhaut V., Maertens L., De Lange L., Marchal L., Nezer C., De Baere S., Croubels S., Daube G., Dewulf J. (2015). Effects of Xylo-Oligosaccharides on Broiler Chicken Performance and Microbiota. Appl. Environ. Microbiol..

[52] Cordero G., Kim J.C., Whenham N., Masey-O’Neill H., Srinongkote S., González-Ortiz G. (2019). 163 Xylanase and fermentable xylo-oligosaccharides improve performance in grower-finisher pigs fed a corn-soybean meal based diet. J. Anim. Sci..

[53] den Besten G., van Eunen K., Groen A.K., Venema K., Reijngoud D.-J., Bakker B.M. (2013). The role of short-chain fatty acids in the interplay between diet, gut microbiota, and host energy metabolism. J. Lipid Res..

[54] Pouteau E., Rochat F., Jann A., Meirim I., Sanchez-Garcia J.-L., Ornstein K., German B., Ballèvre O. (2008). Chicory increases acetate turnover, but not propionate and butyrate peripheral turnovers in rats. Br. J. Nutr..

[55] Melo-Duran D., Gonzalez-Ortiz G., Sola-Oriol D., Martinez-Mora M., Perez J.F., Bedford M.R. (2019). Relationship between peptide YY, cholecystokinin and fermentation products in fasted, re-fed and ad libitum fed broiler chickens. Anim. Feed Sci. Technol..

[56] Adedokun S.A., Olojede O.C. (2019). Optimizing Gastrointestinal Integrity in Poultry: The Role of Nutrients and Feed Additives. Front. Vet. Sci..

[57] Kheravii S.K., Swick R.A., Choct M., Wu S.B. (2018). Upregulation of genes encoding digestive enzymes and nutrient transporters in the digestive system of broiler chickens by dietary supplementation of fiber and inclusion of coarse particle size corn. BMC Genom..

[58] Eeckhaut V., Van Immerseel F., Croubels S., De Baere S., Haesebrouck F., Ducatelle R., Louis P., Vandamme P. (2011). Butyrate production in phylogenetically diverse Firmicutes isolated from the chicken caecum. Microb. Biotechnol..

[59] Onrust L., Ducatelle R., Van Driessche K., De Maesschalck C., Vermeulen K., Haesebrouck F., Eeckhaut V., Van Immerseel F. (2015). Steering Endogenous Butyrate Production in the Intestinal Tract of Broilers as a Tool to Improve Gut Health. Front. Vet. Sci..

[60] Scott K.P., Martin J.C., Duncan S.H., Flint H.J. (2014). Prebiotic stimulation of human colonic butyrate-producing bacteria and bifidobacteria, in vitro. FEMS Microbiol. Ecol..

[61] Upadhyaya B., McCormack L., Fardin-Kia A.R., Juenemann R., Nichenametla S., Clapper J., Specker B., Dey M. (2016). Impact of dietary resistant starch type 4 on human gut microbiota and immunometabolic functions. Sci. Rep..

[62] Biddle A., Stewart L., Blanchard J., Leschine S. (2013). Untangling the Genetic Basis of Fibrolytic Specialization by Lachnospiraceae and Ruminococcaceae in Diverse Gut Communities. Diversity.

[63] Dou S., Gadonna-Widehem P., Rome V., Hamoudi D., Rhazi L., Lakhal L., Larcher T., Bahi-Jaber N., Pinon-Quintana A., Guyonvarch A. (2017). Characterisation of Early-Life Fecal Microbiota in Susceptible and Healthy Pigs to Post-Weaning Diarrhoea. PLoS ONE.

[64] Van Immerseel F., De Buck J., Pasmans F., Velge P., Bottreau E., Fievez V., Haesebrouck F., Ducatelle R. (2003). Invasion of Salmonella enteritidis in avian intestinal epithelial cells in vitro is influenced by short-chain fatty acids. Int. J. Food Microbiol..

[65] Vermeulen K., Verspreet J., Courtin C.M., Haesebrouck F., Ducatelle R., Van Immerseel F. (2017). Reduced particle size wheat bran is butyrogenic and lowers Salmonella colonization, when added to poultry feed. Vet. Microbiol..

